# Solidarity for the mpox outbreak: A call for a unified global response

**DOI:** 10.4102/jphia.v15i1.784

**Published:** 2024-10-04

**Authors:** Banda Khalifa, Morẹ́nikẹ́ O. Foláyan, Nicaise Ndembi

**Affiliations:** 1 Johns Hopkins Bloomberg School of Public Health, Baltimore, United States of America; 2 Department of Child Dental Health, Obafemi Awolowo University, Ile-Ife, Nigeria; 3 Africa Centres for Disease Control and Prevention (Africa CDC), Addis Ababa, Ethiopia

## Introduction

Mpox poses a significant public health threat, particularly as the world continues to grapple with the lingering profound impact of the COVID-19 pandemic on Africa: the pandemic led to a contraction in the region’s economic growth by 3.2% in 2020.^1^ Additionally, it increased the number of people living below the poverty line, significantly reduced government health expenditure and constrained Africa’s progress toward attaining the 2030 Sustainable Development Goals (SDGs).^2^ The pandemic also placed enormous pressure on health systems, highlighting the fragility of the continent’s health infrastructure.^3^ Just as Africa was beginning to strengthen its emergency response measures, including surveillance, prevention, clinical care and efforts to enhance vaccine production and access to build more robust and resilient health systems, the continent now faces a new health crisis: the resurgence of mpox. With over 40 874 cases and 1512 deaths reported across 15 African Union (AU) member states since 2022,^4^ the yet to be resilient health systems are again out under strain. The fragile health systems are now tasked with managing yet another significant health crisis that demands immediate attention.

On 13 August 2024, the Africa Centres for Disease Control and Prevention (Africa CDC) declared the ongoing mpox outbreak a Public Health Emergency of Continental Security (PHECS).^5^ The following day, the World Health Organization (WHO) escalated the situation to a Public Health Emergency of International Concern (PHEIC).^6^ These announcements came on the heels of the escalating number of people affected by mpox, reports of cases beyond the borders of countries in which the disease is endemic and the emergence of a new strain of the virus – clade 1b, detected in neighbouring African countries as well as Sweden and Thailand.^7^ With 17 541 cases and 517 deaths reported in 2024 alone, a number exceeding the report of the same period in 2023 by 160%, the outbreak demands swift and decisive global action.^5^

## The case for global solidarity

The mpox outbreak, much like COVID-19, underscores the critical need for coordinated international responses to global health crises. In an interconnected world, no country is truly safe until every country is protected. This reality demands that global collaboration be prioritised over isolationism, recognising that health emergencies that transcend borders can be effectively controlled only through collective action.

The early stages of the COVID-19 pandemic were marred by delays in global cooperation, resulting in devastating consequences. By March 2021, high-income countries, representing just 16% of the world’s population, had secured over 60% of the global vaccine supply. This left many low-income nations with insufficient access to life-saving doses, exacerbating the crisis and prolonging the pandemic.^8^ The mpox outbreak must not follow this trajectory. Rapid, equitable distribution of vaccines, diagnostics and treatments is imperative, supported by transparent data sharing and robust international cooperation. The global community cannot afford a repeat of COVID-19 by allowing vaccine nationalism to influence the global mpox response negatively.

In addition, the ethical responsibility of high-income nations to support lower-income countries transcends charity; it is an obligation for global stability and security. This global obligation is centred on the need for high-income countries to prioritise the welfare of low- and middle-income countries (LMICs) as a moral obligation to prevent wealth disparities from determining access to life-saving medical care during global pandemics like the current mpox outbreaks. This obligation is rooted in the duty to rectify the historical harms inflicted by colonial powers on former colonies. In addition, there is a negative duty to avoid perpetuating a global institutional order that remains unjust when it can be reformed by adopting a model of vaccine distribution that ensures equitable access for all.^9^ The current call for global solidarity is for fairness and global stability that transients from a charity-based approach to one rooted in equity and justice. It is a call to ensure Africa has the resources and support necessary for self-reliance to control the mpox outbreaks on the continent and institute effective pandemic preparedness programmes.

## Strengthening global solidarity in response to the mpox outbreak

The mpox outbreak, much like previous global health crises, demands an urgent and united international response. Equitable distribution of mpox vaccines to low-income countries is a moral and practical necessity. High-income countries must actively share vaccine doses with regions hardest hit by the outbreak. This requires immediate logistical coordination and international funding to support vaccine deployment in under-resourced areas. Such efforts are crucial to prevent the mpox outbreak from evolving into another protracted global health emergency.

In addition, increased funding for global health initiatives that support pandemic preparedness, disease surveillance and health system strengthening in LMICs is also crucial. Establishing a dedicated international mpox response fund, akin to the United States Mpox Vaccine Equity Pilot Program (MVEPP),^10^ is a possible mechanism for directing resources to where they are most needed during the current crisis. This could help ensure that LMICs receive timely access to vaccines, treatments and diagnostics, enabling a more equitable and effective global response.

A legally binding framework that promotes equitable distribution of critical resources during global health emergencies is needed. Such a framework could promote fairness and fortify global trust and cooperation in managing health emergencies. One effort in this direction is the proposed Pandemic Treaty under the WHO, which aims to ensure that all countries have access to necessary resources such as vaccines, treatments and diagnostics, thereby preventing disparities and fostering a unified global response to future pandemics. The negotiations on a global pandemic agreement by the members of the World Health Assembly are expected to be completed by June 2025 at the latest.^11^ Such a treaty is needed for a time like this. It can support strengthening Africa’s research and development capacity to ensure a rapid and effective response to emerging health threats. In addition, it can support technology transfer and infrastructure development that can enhance the decentralisation and expansion of pharmaceutical and vaccine manufacturing capabilities in Africa. Building local manufacturing capacity reduces reliance on a few global suppliers, mitigates supply chain disruptions and ensures that vaccines and treatments are produced closer to where they are most needed.

The global spread of mpox, a recognised endemic disease in Africa since 1970, is a call to proactively prevent further escalation of such diseases and protect vulnerable populations worldwide. Africa, home to significant populations of children, women and marginalised communities, often bears the brunt of health crises.^12^ Moreover, Africa hosts over 100 endemic diseases, with the social, economic, environmental and ecological factors that elevate the risk for emerging infectious diseases being particularly prevalent in many African countries.^13,14^ Continued global solidarity is imperative for effectively managing and controlling these diseases and building Africa’s resilience to handle its health challenges and reduce the risk of future global outbreaks. The mpox outbreak in Africa presents yet another opportunity to embrace this call for solidarity, allowing the world to emerge stronger, more resilient and more unified than ever before.
